# Descending colon cancer in a patient with situs inversus totalis: a case report and literature review

**DOI:** 10.1093/jscr/rjab568

**Published:** 2022-03-26

**Authors:** Long Cheng, Yi-Guo Feng, Lin He, Jie-Bin Xie, Cen-Ji Zhou, Jian-Jun Liu, Pan Wang

**Affiliations:** Department of Gastrointestinal Surgery, Affiliated Hospital of North Sichuan Medical College, Nanchong, China; Sichuan Key Laboratory of Medical Imaging, Nanchong, China; Department of Gastrointestinal Surgery, Affiliated Hospital of North Sichuan Medical College, Nanchong, China; Sichuan Key Laboratory of Medical Imaging, Nanchong, China; Department of Gastrointestinal Surgery, Affiliated Hospital of North Sichuan Medical College, Nanchong, China; Sichuan Key Laboratory of Medical Imaging, Nanchong, China; Department of Gastrointestinal Surgery, Affiliated Hospital of North Sichuan Medical College, Nanchong, China; Department of Gastrointestinal Surgery, Affiliated Hospital of North Sichuan Medical College, Nanchong, China; Department of Gastrointestinal Surgery, Affiliated Hospital of North Sichuan Medical College, Nanchong, China; Department of Gastrointestinal Surgery, Affiliated Hospital of North Sichuan Medical College, Nanchong, China; Sichuan Key Laboratory of Medical Imaging, Nanchong, China

## Abstract

Situs inversus totalis (SIT) is a congenital disorder of anatomical position, and the operation of patients with total visceral inversion often brings great challenges to surgeons. Although there have been previously documented on patients with SIT and colonic cancer, this is the first case report of descending colon cancer in patient with SIT. The current report presents a case of a 67-year-old female patient with descending colon cancer and SIT. After preoperative preparation and discussion, open left hemicolectomy was performed for the patient. The postoperative recovery of the patient was smooth; however, there was a mild lymphatic leakage in the patient, which was cured by conservative treatment for 5 days. The patient was discharged on postoperative Day 10. There was no tumor recurrence or other discomfort in 1 year follow-up period.

## INTRODUCTION

Situs inversus totalis (SIT) is a congenital disorder of anatomical position, which can be inherited by autosomal recessive inheritance [1]. It is characterized by transposition of visceral position of chest and abdominal cavity, which looks like a mirror of normal person, termed ‘mirror man’. SIT is often associated with various congenital anomalies, including congenital heart diseases, renal dysplasia and biliary atresia [2]. Because of the variation of its anatomical position, it greatly increases the challenge of assessment and operation for surgeons. Recently, there have been several reports of SIT patients with ascending colon cancer, transverse colon cancer and sigmoid colon cancer [3–5]. To the best of our knowledge, this is the first case reported in the literature of descending colon cancer in patient with SIT. In this article, we present the anatomical variation of the case and review the literature to discuss the diagnosis and treatment of the patients with SIT.

## CASE PRESENTATION

A 67-year-old female patient was admitted to our hospital in May 2019 due to abdominal pain and distention on the right side of the abdominal cavity for >1 month, and the abdominal pain was relieved after defecation, with no dizziness, fever, bloody stool and other symptoms. One week before her admission, colonoscopy of the patient, outside our hospital, indicated descending colon cancer with slight intestinal stenosis, and the result of pathological examination confirmed colonic adenocarcinoma. The medical history of the case includes SIT for >30 years and diabetes for >3 years via a normal physical examination.

**Figure 1:**
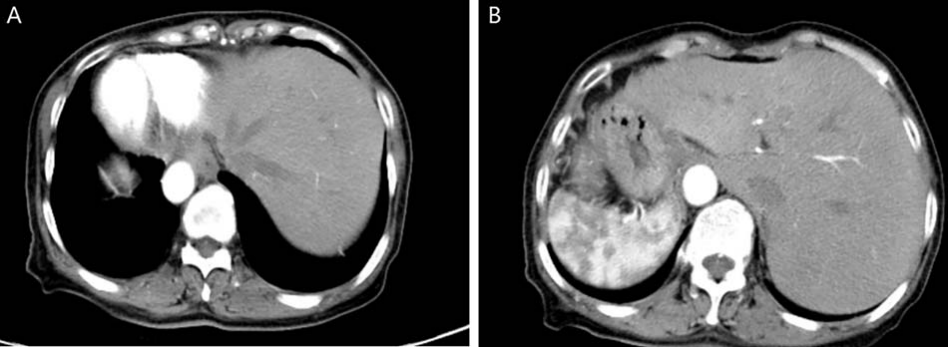
Enhanced CT of the upper abdomen showed that the heart was located on the right side of the thoracic cavity, the liver was located on the left side of the abdominal cavity (**A**), and the stomach and spleen were located on the right side of the abdominal cavity (**B**), confirming SIT.

**Figure 2:**
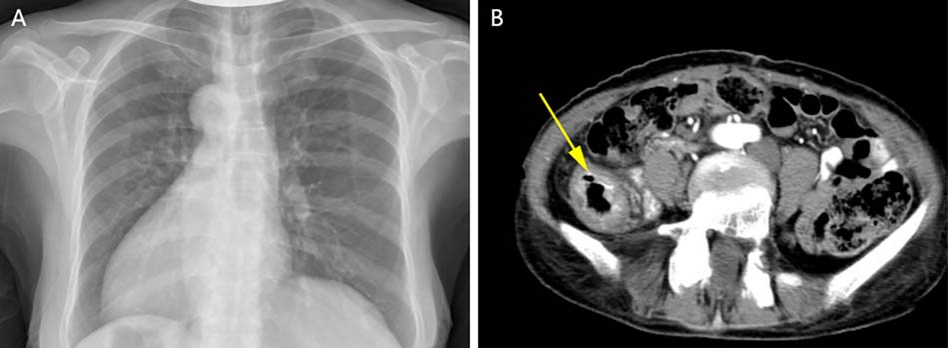
Chest X-ray showed that the heart was on the right side of the chest (**A**). Enhanced CT of the lower abdomen showed thickening of the descending colon with stenosis and enhancement, which suggested that it might be a tumor of the descending colon (**B**, yellow arrow).

A physical examination revealed that the abdomen is flat, without gastrointestinal type and peristaltic wave, and the right lower abdomen has mild tenderness, with no rebound pain and muscle tension, and the mass can not be touched. Serum tumor markers were not elevated (CEA 2.3 ng/ml and CA-199 4.15 ng/ml). The laboratory examination confirmed mild anemia and hypoproteinemia. Abdominal computerized tomography (CT) showed the apex of the heart on the right side, the liver on the left side of the abdominal cavity (Fig. 1A), the stomach and spleen on the right side of the abdominal cavity (Fig. 1B), confirming SIT. In addition, the chest X-ray confirmed the apex of the heart on the right chest (Fig. 2A), and abdominal CT indicated that the tumor (stage T4N0M0) was located in the descending colon (Fig. 2B).

**Figure 3:**
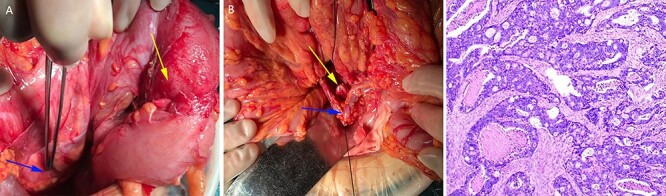
The left ovarian blood veins were significantly thickened (**A**, blue arrow), and the tumor was located in the descending colon (**A**, yellow arrow); the inferior mesenteric blood vessels were absent, the left colon blood vessels (**B**, blue arrow) and the left ureter (**B**, yellow arrow) were marked. Pathological examination showed a moderately differentiated adenocarcinoma (**C**).

After sufficient preoperative preparation and discussion, we decided to perform open radical resection of colon cancer operation for the patient. During surgery, we found that the tumor was located in the lower part of the descending colon (in the right abdominal cavity), the size of the tumor was ~4.0 x 5.0 cm in diameter, the boundary between the tumor and the surrounding tissue was clear and the tumor invaded the whole intestinal wall. Moreover, the patient’s the left ovarian vessels were significantly thickened, especially the veins (Fig. 3A)， and the inferior mesenteric vessels were absent (Fig. 3B). The operation time was 115 min and intraoperative blood loss was 33 ml. Histological examination of the resected specimens revealed a moderately differentiated carcinoma (Fig. 3C). On postoperative Day 1, the laboratory examination showed that white blood cell count was 13 600/mm^3^, and neutrophil cell count was 12 300/mm^3^. These results showed that the leukocytes and neutrophils were significantly increased, and antibiotics were applied to prevent the occurrence of infection in consideration of aseptic inflammatory reaction in the operation area and systemic stress reaction. There was no obvious abdominal pain, chills, fever and other discomfort in the patient after operation. The drainage tube outflow was a pale red liquid (~50 ml/day). The anal exhaust time was on postoperative Day 3 and the patient started a fluid diet. However, on postoperative Day 5, the peritoneal drainage volume had increased to ~500 ml/day and the liquid had become pale yellow, which was confirmed as lymphatic leakage by Sudan III staining (data not shown). Treatment with low-fat diet for 5 days, the drainage fluid of the patient was significantly decreased to ~10 ml/day and then the drainage tube was removed as B-ultrasonography examination showed almost no remaining liquid in the abdominal cavity. The final pathological report of the case showed a moderately differentiated carcinoma and stage T4aN0M0. The patient was discharged on postoperative Day 10 and followed up for 1 year without tumor recurrence or tumor metastasis. No special discomforts and complications were mentioned in the 1 year followed-up period (data not shown).

## DISCUSSION

The whole visceral inversion is a rare variation of human visceral anatomy. It is characterized by the complete bump of the position of the chest and abdomen organs, which is similar to the mirror of normal person, so it is also called ‘mirror man’. SIT is a rare congenital anomaly and the incidence is 1 in 5000–10 000 [1]. The etiology of SIT is considered to be autosomal recessive inheritance, however, it is not clear whether there are other factors leading to its occurrence [6]. Previous reports have found that total visceral inversion is often accompanied by absence of spleen, biliary atresia or intestinal malrotation. Intestinal malrotation is an abnormality of intestinal internal fixation, characterized by the displacement of Treitz ligament [7]. The digestive, circulatory, respiratory and other physiological functions of most patient cases with total visceral inversion are the same as those of normal person. At present, there is no report that total visceral inversion will affect the length of life and quality of life of patients, but cases with this mutation have a higher risk of gastrointestinal cancer [8]. Because of the great variation of its anatomical structure, the diagnosis and treatment of patients are difficult, especially when the patients with total visceral inversion need surgical treatment, which greatly increases the challenge of surgical operation.

To our knowledge, a total of 15 case reports of total visceral inversion with colorectal tumor have been reported (see Table 1), and all of the 16 cases have been successfully operated [3–5, 9–19]. These reports emphasize detailed preoperative examination and perfect surgical plan for patients with SIT. In addition to chest X-ray, laboratory examination and abdominal enhanced CT scan, it is recommended that preoperative patients with SIT also need undergo cardiac ultrasound examination to evaluate cardiac valve pathology or cardiac malformation. If the condition is permitted, preoperative angiography is necessary for patients with total visceral inversion, which is important to determine the variation of anatomical structure. Abdominal 3-dimensional (3D) CT angiography and CT colonography can detect the anatomical structure of patients, especially the situation of colonic blood vessels, which is helpful for surgeons to discover the possible vascular anatomical variation before operation and accurately ligate the corresponding blood supply vessels of tumor as soon as possible in operation, so as to reduce the possibility of intraoperative bleeding and blood metastasis of tumor, and to save operation time. We did not perform 3D CT angiography and CT colonography for this case before operation in consideration of the patient’s economic reasons. Therefore, we chose open radical resection of descending colon cancer for the patient. During the operation procedure, we found that there was an apparent variation in the anatomy of the colon vessels in this case, and there were no definite inferior mesenteric vessels. Due to the anatomical variation of human viscera, surgeons should pay more attention to the protection of adjacent organs during operation and avoid side injury. In the present report, the left ovarian blood vessels in this case were obviously thickened, which was easy to be mistaken as inferior mesenteric vessels, resulting in side injury. The patient had slight lymphatic leakage after operation, which was completely cured by conservative treatment, such as low-fat diet treatment. There was no special discomfort and complications in the follow-up of the patient.

**Table 1:** Summary of cases with SIT combined with colorectal cancer

Author	Year	Location	Other malformation	Complication	Blood loss (ml)	Operating time (min)
Fujiwara *et al.* [3]	2007	Ascending colon	No	None	60	191
Huh JW *et al.* [4]	2010	Lower rectum	No	None	<120	250
Kim WK *et al.* [5]	2011	Transverse And sigmoid colon	No	None	–	–
Kim HJ *et al.* [9]	2011	Ascending colon	No	None	Minimal	119
Lee JM *et al.* [10]	2013	Ascending colon	No	None	Minimal	120
Sumi Y *et al.* [11]	2013	Transverse colon	No	None	230	402
Hirano Y *et al.* [12]	2015	Cecal	No	None	Minimal	125
Yaegas-hi M *et al.* [13]	2015	Sigmoid colon	No	None	13	189
Sasaki K *et al.* [14]	2017	Ascending colon	No	None	10	109
Takeda T *et al.* [15]	2018	Sigmoid colon	No	None	Low	195
Kojima Y *et al.* [16]	2019	Ascending colon	No	None	20	237
Karabay O *et al.* [17]	2019	Sigmoid colon	No	None	–	–
Takeda T *et al.* [18]	2019	Sigmoid colon	No	None	Low	195
Chen W *et al.* [19]	2020	Sigmoid colon	No	None	–	120

In conclusion, this paper reports the descending colon cancer in case with SIT for the first time and reviews the literature. For the diagnosis and therapy of patients with SIT combined with gastrointestinal diseases, the possibility of visceral inversion should always be considered. Moreover, the operation can be performed safely by laparoscopy in center of medical institutions with mature technology and equipment. With the continuous advancement of technology, there will be a further recognization in patients with SIT associated with cancer.

## AUTHORS’ CONTRIBUTIONS

Cheng L, Feng YG and Wang P wrote the manuscript; Cheng L, Feng YG, He L, Xie JB, Zhou CJ, Liu JJ and Wang P diagnosed and treated; all authors approved the final version and took responsibility for its final content.

## CONFLICT OF INTEREST STATEMENT

All authors declare that they have no competing interests.

## FUNDING

This work was supported by the program of strategic cooperation in science and technology of Nanchong City of China (18SXHZ0110).

## References

[1] Iwamura T, Shibata N, Haraguchi Y, Hisashi Y, Nishikawa T, Yamada H, et al. Synchronous double cancer of the stomach and rectum with situs inversus totalis and polysplenia syndrome. J Clin Gastroenterol. 2001;33:148–53. 11468444 10.1097/00004836-200108000-00012

[2] Spoon JM . Situs inversus totalis. Neonatal Netw. 2001;20:59–63. 10.1891/0730-0832.20.1.63 12143842

[3] Fujiwara Y, Fukunaga Y, Higashino M, Tanimura S, Takemura M, Tanaka Y, et al. Laparoscopic hemicolectomy in a patient with situs inversus totalis. World J Gastroenterol. 2007;13:5035–7. 17854150 10.3748/wjg.v13.i37.5035 PMC4434631

[4] Huh JW, Kim HR, Cho SH, Kim CY, Kim HJ, Joo JK, et al. Laparoscopic total mesorectal excision in a rectal cancer patient with situs inversus totalis. J Korean Med Sci. 2010;25:790–3. 20436720 10.3346/jkms.2010.25.5.790 PMC2858843

[5] Kim YW, Ryu H, Kim DS, Kim IY. Double primary malignancies associated with colon cancer in patients with situs inversus totalis: two case reports. World J Surg Oncol. 2011;9:109. 21943483 10.1186/1477-7819-9-109 PMC3191476

[6] Goi T, Kawasaki M, Yamazaki T, Koneri K, Katayama K, Hirose K, et al. Ascending colon cancer with hepatic metastasis and cholecystolithiasis in a patient with situs inversus totalis without any expression of UVRAG mRNA: report of a case. Surg Today. 2003;33:702–6. 12928850 10.1007/s00595-002-2567-y

[7] Zhu H, Yang K, Hu JK. Gastrectomy for gastric carcinoma with situs inversus totalis: case report and literature review. Hippokratia. 2015;19:360–2. 27703309 PMC5033149

[8] Galiatsatos P, Kasprzak L, Chong G, Jass JR, Foulkes WD. Multiple primary malignancies in a patient with situs ambiguous. Clin Genet. 2006;69:528–31. 16712706 10.1111/j.1399-0004.2006.00622.x

[9] Kim HJ, Choi GS, Park JS, Lim KH, Jang YS, Park SY, et al. Laparoscopic right hemicolectomy with D3 lymph node dissection for a patient with situs inversus totalis: report of a case. Surg Today. 2011;41:1538–42. 21969158 10.1007/s00595-010-4530-7

[10] Lee JM, Jung SY. Laparoscopic hemicolectomy in ascending colon cancer with situs Inversus Totalis. J Laparoendosc ADV S, Videoscopy 2013;23.

[11] Sumi Y, Tomono A, Suzuki S, Kuroda D, Kakeji Y. Laparoscopic hemicolectomy in a patient with situs inversus totalis after open distal gastrectomy. World J Gastroenterol Surg. 2013;5:22–6. 10.4240/wjgs.v5.i2.22 PMC3600568 23515492

[12] Hirano Y, Hattori M, Douden K, Hashizume Y. Single-incision laparoscopic surgery for colon cancer in patient with situs inversus totalis: report of a case. Indian J Surg. 2015;77:26–8. 25972634 10.1007/s12262-014-1075-9 PMC4425779

[13] Yaegashi M, Kimura T, Sakamoto T, Sato T, Kawasaki Y, Otsuka K, et al. Laparoscopic Sigmoidectomy for a patient with situs Inversus Totalis: effect of changing operator position. Int Surg. 2015;100:638–42. 25875545 10.9738/INTSURG-D-14-00217.1 PMC4400931

[14] Sasaki K, Nozawa H, Kawai K, Hata K, Kiyomatsu T, Tanaka T, et al. Laparoscopic hemicolectomy for a patient with situs inversus totalis: a case report. Int J Surg Case Rep. 2017;41:93–6. 29055878 10.1016/j.ijscr.2017.10.011 PMC5651547

[15] Martínez ML, Redondo PV, Gatica JC, Angel JMR. Laparoscopic hemicolectomy for a patient with situs inversus totalis and colorectal cancer. Journal of Coloproctology 2017;147–51.

[16] Kojima Y, Sakamoto K, Tomiki Y, Sugimoto K, Okazawa Y, Makino Y. Laparoscopic right colectomy for a patient with situs inversus totalis. Journal of Surgical Case Reports 2019;3:1–3. 10.1093/jscr/rjz080 PMC6439510 30949333

[17] Karabay O, Gurbuz B, Zenger S, Balik E, Bugra D. Laparoscopic colon resection in patients with situs inversus totalis: is it the same operation as in patients without situs inversus totalis? J Minim Access Surg. 2019;15:68–70. 29737309 10.4103/jmas.JMAS_13_18 PMC6293681

[18] Takeda T, Haraguchi N, Yamaguchi A, Uemura M, Miyake M, Miyazaki M, et al. Laparoscopic sigmoidectomy in a case of sigmoid colon cancer with situs inversus totalis. Asian J Endosc Surg. 2019;12:111–3. 29601667 10.1111/ases.12483 PMC6585653

[19] Chen W, Liang JL, Ye JW, Luo YX, Huang MJ. Laparoscopy-assisted resection of colorectal cancer with situs inversus totalis: a case report and literature review. World J Gastrointest Endosc. 2020;12:310–6. 32994862 10.4253/wjge.v12.i9.310 PMC7503618

